# DNA Mimics for the Rapid Identification of Microorganisms by Fluorescence *in situ* Hybridization (FISH)

**DOI:** 10.3390/ijms9101944

**Published:** 2008-10-20

**Authors:** Laura Cerqueira, Nuno F. Azevedo, Carina Almeida, Tatiana Jardim, Charles William Keevil, Maria J. Vieira

**Affiliations:** 1 IBB - Institute for Biotechnology and Bioengineering, Centre of Biological Engineering, Universidade do Minho, Campus de Gualtar 4710-057, Braga, Portugal. E-Mails: lauracerqueira66@iol.pt (L. C.); carinaalmeida@deb.uminho.pt (C. A.); tatianajardim@gmail.com (T. J.); mjv@deb.uminho.pt (M. V.); 2 Environmental Healthcare Unit, School of Biological Sciences, University of Southampton, Bassett Crescent East, Southampton SO16 7PX, UK. E-Mail: C.W.Keevil@soton.ac.uk (N. A.)

**Keywords:** FISH, DNA mimics, PNA, LNA, molecular diagnostics

## Abstract

Fluorescence *in situ* hybridization (FISH) is a well-established technique that is used for a variety of purposes, ranging from pathogen detection in clinical diagnostics to the determination of chromosomal stability in stem cell research. The key step of FISH involves the detection of a nucleic acid region and as such, DNA molecules have typically been used to probe for the sequences of interest. However, since the turn of the century, an increasing number of laboratories have started to move on to the more robust DNA mimics methods, most notably peptide and locked nucleic acids (PNA and LNA). In this review, we will cover the state-of-the-art of the different DNA mimics in regard to their application as efficient markers for the presence of individual microbial cells, and consider their potential advantages and pitfalls. Available PNA probes are then reassessed in terms of sensitivity and specificity using rRNA databases. In addition, we also attempt to predict the applicability of DNA mimics in well-known techniques attempting to detect *in situ* low number of copies of specific nucleic acid sequences such as catalyzed reporter deposition (CARD) and recognition of individual genes (RING) FISH.

## 1. FISH for Microbial Detection

Conventional *in situ* hybridization (ISH) is based on the annealing of DNA or RNA molecules to a specific target sequence within a cell. To identify that a successful ISH has occurred, different detection methods have been devised. For instance, in chemiluminescent *in situ* hybridization (CISH), the nucleic acids are coupled with an enzyme such as soybean peroxidase [1]. This enzyme will then recognize the presence and cleave a chemiluminescent substratum, thereby releasing light that can be captured. More commonly, nucleic acids are attached to a fluorescent label in a technique named as fluorescence *in situ* hybridization (FISH) [2]. Typical labels include cyanine (e. g. Cy3 and Cy5) and fluorescein molecules, but a novel generation of fluorophore families that includes de Alexa Fluors and of nanosized crystal particles named quantum dots [3, 4], is gaining widespread acceptance. The advantages of these new dyes are based on an increased photostability and brightness. Due to serious methodological problems that affect the robustness of RNA FISH (even though RNA ISH is quite common to target gene expression), hybridization generally occurs employing DNA molecules. FISH is rapidly becoming one of the most well-established molecular biology techniques, with applications on pathogen detection in clinical samples for patient management [5, 6], identification of novel biomarkers for cancer progression [7, 8], characterization of communities structure and diversity of natural habitats [2, 9, 10], determination of genes presence and expression [11, 12] and even chromosomal stability in stem cell research [13], among many others. In this review, we will focus exclusively on the recent developments of DNA mimics for the identification of microorganisms.

Prior to the analysis of the hybridization results by epifluorescence microscopy or eventually flow cytometry, the FISH method usually comprises three steps – fixation, hybridization and washing. The fixation step involves the application of chemical fixatives, such as formalin, paraformaldehyde and ethanol that are very commonly used in bacterial and human cells [6, 14, 15]. During the hybridization step, temperature, pH, ionic strength and formamide concentrations are all well defined to guarantee that the probe accesses and hybridizes with the target sequence. The washing step ensures that all loosely bound or unbound labelled probes are removed from the sample, hence providing specificity to the detection. After optimization of the hybridization method, it is expected that the probes will only recognize their complementary sequence, hence providing specificity to the method. In general, the probes used for FISH identification target a sequence of the 16S ribosomal RNA in members of the Bacteria or Archaea domain, or the 18S rRNA in members of Eucaryota. The choice of rRNA as a target molecule is related to the abundance of these structures in the cell and to their use as a phylogenetic marker [15, 16]. Ribosome numbers in a single cell range from 10^2^–10^3^ for *Mycobacterium tuberculosis* to 10^4^–10^5^ for *Escherichia coli* [16], which implies that the observed fluorescence intensity is the result of multiple probe labels and is related to ribosomal content [17, 18]. As such, low signal intensity might result from low rRNA content in cells [18].

In spite of the recent surge in the application of DNA FISH, all of those who have worked with this method are aware that not everything is straightforward in probe design and protocol development. For a start, cell membranes are not always permeable to DNA probes. Consequently, pre-treatment with lysozyme or other proteolytic enzymes may be required, particularly for Gram-positive bacteria [15, 19]. Furthermore, rRNA accessibility due to ribosomal secondary structure might imply increased hybridization times of up to 96 hours [20], whereas the degradation of the probe by proteases or endonucleases of living cells may also constitute an obstacle to the implementation of this method [21, 22]. Finally, there have also been concerns about the ability of the method to discriminate sequences with single-base mismatches, hence affecting the specificity [23]. Interestingly, the reason why so many DNA probes failed to provide bright signals has been recently related to low affinity between the probe and target, i.e. to the overall Gibbs free energy change (ΔG° overall) involving the rRNA and DNA interactions during FISH [24]. A strategy involving the elongation of DNA probes has subsequently been shown to increase affinity and hence improve the brightness of probes with a ΔG°overall below the threshold of 10–13.5 kcal/mol [20, 24].

**Figure 1.**
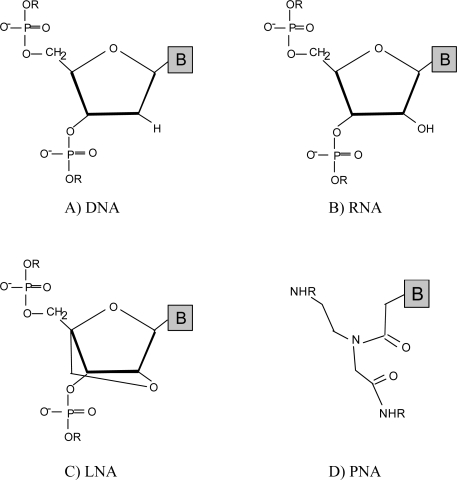
Chemical structures of DNA, RNA and of the DNA mimics, locked nucleic acid (LNA) and peptide nucleic acid (PNA).

With all the problems that have been described associated with DNA FISH, it is no wonder that researchers started to search for alternatives to improve the robustness of this method. The solution appears to have arrived in the form of nucleic acid analogues [25, 26], a new class of molecules that has been originally explored for the regulation of gene expression [27, 28], but that have also found a niche of application on the FISH arena. This new class of molecules is better known as DNA mimics (Figure 1).

## 2. Emergence of DNA Mimics

DNA mimics are emerging as very promising molecules for cell detection in environmental and clinical samples. In fact, only two years after the description of the FISH technique by DeLong *et al.* [17], Nielsen and co-workers published the design and synthesis of peptide nucleic acids (PNA), one of the first DNA analogues to be published [25]. The value of PNA probes for clinical diagnostics has been very recently established by Forrest *et al*. [29, 30]. When applying PNA FISH methods for the rapid differentiation of *S. aureus* from coagulase-negative staphylococci (CoNS) and also for the identification of *C. albicans* in blood cultures, it was determined that there was a significant reduction in median length of hospital stay and a trend towards less antibiotics usage. Consequently, the application of both methods was found to contribute to a decrease in hospital costs per patient and the assay on *C. albicans* has been recently cleared by the US FDA as an *in vitro* diagnostic kit for identification of yeast directly from positive blood cultures [31].

The number of other DNA mimics that have been reported to potentially have application on FISH procedures has in the meantime risen exponentially. The most common, besides PNA, are locked nucleic acid (LNA) [32] and 2’-O-methyl (2’-OMe) RNA [22], but other PNA or LNA-modified molecules have also appeared very recently [e.g. 33, 34–36]. Of these, the two mimics that have been applied for the identification of microorganisms are PNA and LNA [15, 37, 38]. These and other DNA mimics, along with their most relevant characteristics and applications, are described next.

### 2.1. Peptide nucleic acids (PNA)

PNAs were introduced in FISH studies for the detection of microorganisms during the late 1990s [38–41]. In this DNA mimic, the negatively charged sugar-phosphate backbone of DNA is replaced by a neutral polyamide backbone composed of N-(2-aminoethyl) glycine units (Figure 1) [25]. Because of PNA chemical configuration, the nucleobases are practically positioned in the same place and within the same distance as it occurs to the natural DNA. Consequently, PNA can hybridize with complementary DNA or RNA sequences [23, 42].

The lack of electrostatic repulsion, due to the non-charged nature of the PNA backbone is perhaps the main reason responsible for its properties, such as the improved thermal stability compared with DNA/DNA duplexes [42, 43], which implies that the melting temperature (Tm) for PNA/DNA duplexes is higher than for DNA/DNA. This increased Tm is useful for synthesizing PNA probes that are shorter than most DNA probes. In fact, sequences of approximately 15 bp have been found to be optimal for PNA probes which contrast with probes of 20–24 bp for DNA. As a consequence, the effect on the Tm of a single-base mismatch in a PNA/DNA hybridization will have much more impact than in a DNA/DNA hybridization. This factor has great influence on the higher specificity that PNA exhibits for DNA sequence detection [44]. In addition, hybridization could be performed efficiently under low salt concentrations [45], a condition that promotes the destabilization of rRNA secondary structures and results in an improved access to target sequences that would be more elusive using conventional FISH [20, 46, 47]. Like for all other synthetic molecules, PNA also denotes an increased resistance to nucleases and proteases [14, 16, 48]. Finally, diffusion through the cell membrane and naturally occurring microstructures such as the EPS biofilm matrix might be easier, even in Gram-positive bacteria, due to the hydrophobic character of PNA as compared to DNA [40]. However, Stender *et al.* refer the importance of checking for self-complementarity in the design of PNA probes since strong hybridization between PNA complementary sequences can occur [16].

The past few years have seen a significant increase in the number of published PNA probes. In Table 1, the PNA probes that have been designed until the present moment have been described, along with several characteristics such as specificity and sensitivity. Specificity and sensitivity are two of the most important parameters to take into account during probe design. Specificity is expressed as the percentage of the number of strains of the microorganism of interest detected by the probe divided by the total number of microbial strains detected by the probe. Sensitivity is calculated as the percentage of the number of strains of the microorganism of interest detected by the probe divided by the total number of strains of the microorganism of interest present in the database [6]. To calculate these parameters, the Ribosomal Database Project II (RDP II) and the National Centre for Biotechnology Information (NCBI) was used to assess 16S rRNA targets [49, 50], whereas only the NCBI database was used for 18S, 23S and 26S targets. The accuracy of these results is obviously related to the quality and quantity of the sequences deposited in the database, and will vary with time as databases are being constantly updated.

As expected, the large majority of sequences present in Table 1 present a very high (>90%) theoretical specificity and sensitivity. However, some of the probes do present values that are far from expected. One of the explanations is that some probes might have been designed to detect a specific strain of the microorganism of interest. For instance, Azevedo *et al.* designed a probe with 100% specificity but only 24% sensitive [51]. However, the primary aim of the researchers was to detect a specific *H. pylori* strain that was inoculated in a biofilm system in an *in vitro* study. It was therefore important to have very high specificity, but sensitivity (as defined here) was not that relevant in this case as long as the strain of interest was detected. Other possible explanation is that when the probes were originally designed, the number of available sequences in the databases was lower. In fact, with the increasing number of sequences available, it is easy to foresee a scenario where hardly any probe will be 100% specific. The criterion for the choice of the correct probe should therefore fall on the possibility of finding the non-target microorganisms in the samples that will be analyzed for the microorganism of interest.

Peptide nucleic acids are clearly the most advanced technology in respect to applications in FISH, and the robustness of the method implied that some of the probes are now commercially available to perform *in vitro* diagnostic tests (http://www.advandx.com). The synthesis of custom PNA probes is accessible via a South Korean company named Panagene (http://www.panagene.com).

### 2.2. Locked nucleic acids (LNA)

Locked nucleic acid (LNA) is a synthetic RNA derivative in which the ribose is linked to a methylene bridge between 2’-oxigen and 4’-carbon atoms, *i.e.* formed with 2’-O, 4’-C-methylene-ß-D-ribofuranosyl nucleotides (Figure 1) [32, 52]. The connection encloses the sugar, which is responsible for the new conformation that is preferable for the formation of hybrids with complementary DNA or RNA sequences [53]. In fact, it has been shown by thermal studies that DNA duplexes containing LNA residues have the ability to increase the melting temperature between 2 °C and 10 °C per single LNA nucleotide incorporation [26, 54]. Wahlestedt *et al.* demonstrated that LNA oligonucleotides have high *in vivo* efficacy, and hence are very useful in functional genomics and therapeutic applications [28]. As such, these oligonucleotides have so far found more utility in antisense studies rather in FISH methodologies [26, 28].

Typically, FISH probes incorporate only few LNA nucleotides into a DNA strand, compensating the increased affinity of the new strand by a decrease in the number of base pairs used on the probe. Due to strong thermal stability, LNA may self-anneal, being difficult to design, but a web site is available with a help designing tool (www.exiqon.com) [52]. As for PNA, it is important to design LNA probes without extensive self-complementary sequences or applying chimeric LNAs. Equally, this DNA mimic is not susceptible to nucleases, which means that it is optimal when applied in living cell protocols [55].

LNA technology is only now providing the first steps towards application for rapid identification of microorganisms [37]. Nevertheless, applications of LNA in FISH technology for the detection of human miRNA and mRNA [56, 57], together with studies that report increased fluorescence intensities of probes after the substitution of DNA bases by LNA bases [37, 58], indicate that this technology also holds great promise.

### 2.3. Other mimics

2’-OMe RNA, 2’-deoxy-2’-fluoro-ß-d-ribonucleic acid (2’-F RNA) and morpholinos are three other common backbone modified structures that might be important for the development of future *in situ* hybridization procedures. Adding to these, numerous PNA and LNA-based molecules have been developed. Due to their synthetic nature, they all possess increased resistance to enzymes such as nucleases.

2’-OMe RNA probes have demonstrated high affinity, increased specificity and capacity to bind RNA target sequences [59, 60]. In fact, these probes form more stable complexes with complementary RNA sequences than natural DNA and RNA oligonucleotides [61]. This efficient hybridization combined with its chemical structure may lead to the utilization of these polymers in many antisense applications [62, 63]. Even though 2’-OMe RNA probes have never been employed in micro-organisms, they have already found application for RNA detection in living eukaryotic cells [60, 64].

The chemical structure of 2’-F RNA is very similar to that of RNA, where the 2’-OH group is replaced by a fluorine [21]. Hybridization analysis demonstrated that 2’-F RNA hybridized with greater affinity to RNA than 2’-OMe RNA [65, 66]. In 2’-F RNA the Δ Tm increases the more 2’-F RNA bases are incorporated in the probe, i.e., the bigger the 2’-F RNA sequence is, the higher the Tm. However, applications in FISH have been hindered by the difficulty and high cost of synthesis of this molecule.

Morpholinos [e.g. 67, 68–71], PNA-based backbones or monomers such as trans-4-hydroxy-*N*-acetylpyrrolidine-2-phosphonate (PHypNAs) and trans-4-hydroxy-l-proline (HypNA-pPNAs) [72–74] and LNA-based molecules such as phosphorothiolate-LNA and 2’-thio-LNA [75–79] are other chemically synthesised molecules with similar structures to that of DNA/RNA. Up until now, they have been primarily tested for antisense therapy, but their structure might also lead for applications in FISH procedures. It is therefore to be expected that the future will bring a range of new molecules with capacity of detecting cells by FISH, bringing possible benefits to the robustness of the technique.

## 3. Conclusions and Future Work

Before the appearance of DNA mimics, FISH techniques employed natural DNA molecules to perform hybridizations. By now, DNA mimics have unequivocally proved that they might contribute to improve the robustness of the method. Their main advantages include resistance to the attack of enzymes due to their synthetic nature; higher affinity for RNA sequences with a lower number of base pairs, which leads not only to more successful hybridizations but also to easier discrimination of single-base mismatches; and for certain mimics such as PNA, hybridization under low salt conditions allow unrestricted access to the full sequence of rRNA. This begs the question: Why have DNA mimics failed so far to impose themselves over standard DNA methods? The answer is threefold: First of all, not all microbiology laboratories are aware of these technologies, and when they are, it is not always easy to identify the companies or research groups that might provide you with the desired probe and know-how; Secondly, most of the probes have costs many times higher than those associated with DNA probes; Finally, the status of research for DNA probes is many years ahead than that of most DNA mimics. As a consequence, there is a general lack of information on some basic thermodynamic properties. For instance, whereas the estimation of hybridization temperature is quite straightforward for DNA probes in different hybridization solutions [80, 81], all other mimics, arguably with the exception of PNA [82], have little data available. Before imposing DNA mimics-based procedures as the methods of excellence for the whole-cell detection of microorganisms in different settings, these issues need addressing.

In the future, the robustness of DNA mimics methods might improve the application of multiplex procedures, where various species are targeted with different probes at the same time. In fact, Perry-O’Keefe *et al.* developed a new PNA FISH procedure for the simultaneous detection of both Gram-negative and Gram-positive species, something never accomplished using DNA probes [43]. The use of DNA mimics to perform FISH in living cells could be another major area of application but it also appears to be a trickier one, because of all the deleterious processes that the cells undergo during the fixation, hybridization and washing steps. Besides having to support relatively high temperatures, cells will have to support the toxicity of compounds present in the solutions, such as paraformaldehyde and ethanol in the fixation step. So far, hybridization of unfixed cells as been accomplished [83], but there are serious questions over whether these cells remained viable during the whole hybridization process [2]. Nevertheless, with a battery of DNA mimics available, the chances of developing an experiment where the adverse effects of the hybridization process might be overcome are multiplied.

Finally, DNA mimics might also be of use for the detection of low-copy genomic sequences in the chromosome. Using DNA probes, techniques such as catalyzed reporter deposition (CARD) FISH [18], recognition of individual genes (RING) FISH [84] and rolling circle amplification [85] have tried to solve this problem. In CARD FISH, the enzyme horseradish peroxidase is covalently crosslinked to a nucleic acid probe, and if the probe hybridizes, the enzyme will be present inside the cell. When fluorescently-labelled tyramide is added, the enzyme will induce the deposition of these molecules by peroxidase activity. The major shortcoming of this technique is the large size of the enzyme, which means that damaging permeabilization procedures are necessary. Whereas the application of DNA mimics in this method is not expected to significantly affect the entry of the labelled probe, the increased specificity and sensitivity of these analogs might be very useful for the detection of single nucleotide polymorphisms (SNP). So far, only DNA CARD FISH has been attempted, but enzymes coupled with PNA are already accessible via Panagene. RING FISH is based on the hybridization of multiple-labelled probes (one labelled nucleotide every 10–20 bp) that usually have more than two hundred bases length. The probe then anchors other probes that in turn form a network around the cell periphery and emit an halo of fluorescence [84]. As the probe is particularly long, the incorporation of LNA residues would likely improve the specificity and sensitivity of the method. Even though PNA might minimize the membrane permeabilization issue, it is not clear how would such a long PNA probe behave on this method. On rolling circle amplification, the use of DNA mimics as probes seems to be of little use, as the method requires an amplification step mediated by the enzyme DNA-polymerase. Interestingly, the method does use PNA molecules to open the double helix configuration of DNA [85].

Due to their special chemical features, DNA mimics-based methods are expected to play a very important role in cell and gene detection in clinical and environmental samples in the future. The real challenge will be to determine the best nucleic acid for each specific case/method. Even though the chemistry of the molecules will allow us to take some theoretical assumptions, there are many uncertainties on how DNA mimics behave under most conditions. As such, new reliable and systematic screening protocols that compare all these different molecules – both biological and synthetic – should be developed.

## Figures and Tables

**Table 1.** PNA probes already described in the literature together with some of their most important characteristics.

Microorganism	Sequence (5'–3')	Target	GC Content (%)	Hybridization temperature (°C)/solvent concentration	Specificity (%)	Sensitivity (%)	Ref.
**Eucarya**							
*C. albicans*	AGAGAGCAGCATGCA	26S	53	55°C / 30% DMF	96	46	[86]
*C. albicans*	ACAGCAGAAGCCGTG	26S	60	50°C / 30% DMF	91	70	[87]
*C. dubliniensis*	TAGCCAGAAGAAAGG	18S	47	50°C / 30% DMF	100	4	[87]
*D. bruxellensis*	CGGTCTCCAGCGATT	26S	60	50°C / 50% DMF or 50°C / 30% DMF	100	85	[88]
Eucarya	ACCAGACTTGCCCTC	18S	60	55°C/ 0.5% (w/v) SDS 50°C/50% DMF	N.D.	N.D.	[43, 89]
*S. cerevisae*	TTACCGAGGCAAGCT	18S	53	50°C / 50% DMF	N.D.	N.D.	[43]
*T.* subgenus *A*	CGGAACCCAGCCA	18S	69	45°C / 30% DMF	N.D.	N.D.	[90]
*T.* subgenus *A*	GTTGCCACCAGCAGT	18S	60	45°C / 30% DMF	N.D.	N.D.	[90]
*T.* genus *^B^*	GCCCTAACAGGTGTG	18S	60	55°C / 30% DMF	N.D.	N.D.	[90]
*Z. bailii*	CGAGCGAAACGCCTG	18S	67	50°C / 50% DMF	5	50	[89]
**Bacteria**							
*C. coli, C. jejuni* and *C. lari*	CCCTACTCAACTTGT	16S	47	50°C / 30% DMF	100	91	[91]
*E. coli*	TCAATGAGCAAAGGT	16S	40	55°C/ 0.5%(w/v) SDS or 50°C/50% DMF or 57°C / 30% DMF	59	10	[43, 89, 91, 92]
*E. coli*	GCAAAGCAGCAAGCTC	16S	56	50°C/ 0.01% SDS	100	1	[41]
Eubacteria	CTGCCTCCCGTAGGA	16S	67	55°C/ 0.5% (w/v) SDS or 50°C/50% DMF	N.D.	93	[89]
*H. pylori*	GAGACTAAGCCCTCC	16S	60	59°C / 30% DMF	96	90	[6]
*H. pylori*	TAATCAGCACTCTAGCAA	16S	39	55°C / 30% DMF	100	24	[51, 93]
*K. pneumoniae*	CACCTACACACCAGC	23S	60	55°C	100	92	[94]
*L. brevis*	CTCTAAGATTGGCAG	16S	47	50°C / 50% DMF	81	97	[89]
*Legionella* genus	GACGCAGGCTAATCT	16S	53	55°C to 65°C/ 30% DMF	88	68	[95]
*L. pneumophila*	CTGACCGTCCCAGGT	16S	67	55°C to 65°C / 30% DMF	92	100	[95]
*Listeria* genus	CCCCAACTTACAGGC	16S	60	55°C /0.5% SDS	98	91	[96]
*Listeria* genus	AAGGGACAAGCAGT	16S	50	55°C /0.5% SDS	97	97	[96]
*M. avium*	ATGCGTCTTGAGGTC	16S	53	55°C / 40% DMF	95	91	[97]
*M. avium* subsp. *avium* and *M. avium* subsp. *paratuberculosis*	TGCGTCTTGAGGTCC	16S	60	59°C / 30% DMF	100	89	[98]
*M. kansasii*	TATCCCGGTGTGCAG	16S	60	55°C / 40% DMF	57	100	[97]
*M. leprae*	CGCCTTGAAGTCCTA	16S	53	55°C / 40% DMF	100	100	[97]
*M. tuberculosis* complex (MTC) species	GCATCCCGTGGTCCT	16S	67	60°C / 50% DMF	76	100	[97]
*M. tuberculosis* complex (MTC) species	GGTTTTAAGGATTC	16S	40	55°C / 30% DMF	62	100	[38]
Nontuberculous (NTM) mycobacteria species	GCATTACCCGCTGGC	16S	67	55°C / 30% DMF	34	34	[38]
*P. aeruginosa*	CTGAATCCAGGAGCA	16S	53	55°C/ 0.5% (w/v) SDS or 50°C/50% DMF	80	87	[43, 89]
*Salmonella*	TAAGCCGGGATGGC	23S	64	55°C/ 0.5% (w/v) SDS or 50°C/50% DMF	41	60	[43, 89]
*S. aureus*	GCTTCTCGTCCGTTC	16S	60	55°C/ 0.5%(w/v) SDS or 50°C/50% DMF	100	92	[43, 89]

N.D. – Not determined

^A^T. brucei gambiense, T. brucei rhodesiense, T. brucei brucei, T. envasi and T. equiperdum

*^B^*All *Trypanozoon* species
